# 

**Published:** 1909-11-27

**Authors:** 


### BOOKS RECEIVED.

W. Hodge and Co.

"Construction, Equipment, and Management of a General Hospital." By Donald J. Mackintosh, M.B., M.V.O.

J. B. Lippincott Co.

"International Clinics," Vol. III., 19th series.

"How to Feed Children." By Louise E. Hogan.

"A Handbook of Medical Diagnosis." By J. C. Wilson, A.M., M.D.

T. Fisher Unwin.

"Psychotherapy." By Hugo Münsterberg, M.D., etc.

Henry Frowde, Hodder and Stoughton.

"Manual of Surgery," Vols. I. and II. By A. Thomson, F.R.C.S., and A. Miles, F.R.C.S.

John Murray.

"Mosquito or Man." By Sir Rupert Boyce, M.B., F.R.S.

### GIFTS AND BEQUESTS.

MR. L. W. EVANS, of Southport, left £5,000 to the Liverpool Royal Infirmary.

"HOSPITAL SUNDAY" in York this year produced £250 odd as against £213 odd last year.

THE London Throat Hospital, Great Portland Street, has received a legacy of £200 under the will of the late Mr. Joseph John Heal.

THE lease of Dundee Private Hospital expires shortly, and the committee proposes holding a bazaar next year to raise funds in order to secure other and larger premises.

MR. H. J. ALLCROFT, Chairman and Treasurer of the Royal Hospital for Incurables, Putney Heath, having appealed in a letter to the *Times* for a billiard table for the use of the patients, the Duchess of Northumberland has sent a full-sized billiard table for the use of the male patients.

THE British Home and Hospital for Incurables held the half-yearly general meeting of its subscribers at the Home, Streatham, on October 11. Mr. I. Hampton Hall, the Treasurer, announced a debt of £2,000 for the half-year, "which the board had had to incur to keep the institution going."

AT the annual meeting of the Liverpool Hospital Sunday Fund Sir William H. Tate, Bart., offered to guarantee the amount of expenses during the coming year, the sum not to exceed £250. The offer was cordially accepted. This year Mr. George Nicholson has generously paid the expenses. The Committee reports that the receipts from the collections made on Hospital Sunday of 1909 were £6,235 18s. 5d., against £6,423 15s. 4d. in 1908, being a decrease of £187 16s. 11d. The total amount available for distribution was £6,333 12s. 5d.

UNDER the will of Mr. W. Lavers the Western Hospital, Torquay, will receive £5,000 next year.

THE Middlesex Hospital has received a further grant of £200 from the Goldsmiths' Company to the general fund.

THE Earl of Beaconsfield Memorial Cottage Hospital at High Wycombe has received a gift of £600 from Mrs. Wheeler.

MR. JAMES BILHAM left, subject to life interest, £100 each to the East Suffolk Hospital, St. George's Hospital, and the Norfolk and Norwich Hospital.

MISS MARION ELIZABETH WILLCOX, subject to numerous other bequests, left the residue of her estate (estimated at about £30,000) in equal shares to eighteen charities, one of which was the Royal Hospital for Incurables, Putney.

THE following American bequests are reported: In the will of the late Jane G. Phelps the following hospitals were remembered: Presbyterian Hospital of New York, $10,000; Tarrytown Hospital, $5,000; Lincoln Hospital and Home, Manhattan, $5,000. Chicago Charities benefited $382,000 by the will of the late Mrs. Nelson Morris. Of this amount $300,000 is left for the founding of a convalescent hospital or home for children. The Children's Memorial Hospital receives $5,000; Michael Reese Hospital, $10,000; The Chicago Lying-in Hospital and Dispensary, $2,500; The Chicago Home for Incurables, $5,000; and the Chicago-Winfield Sanatorium, $5,000. At the twenty-fifth anniversary of the Montefiore Home in New York the announcement was made of gifts aggregating $101,500. By the will of the late Miss Anna J. Van Reed, of Reading, Pa., the sum of $13,000 is bequeathed to the Medico-Chirurgical Hospital of Philadelphia.

### Appointments.

Dr. A. M. H. Gray, M.R.C.P., has been appointed physician-in-charge of the Skin Department of University College Hospital, London.

Mr. George Coats has been appointed assistant surgeon to the Royal London Ophthalmic Hospital (Moorfields Eye Hospital).

Mr. H. J. Waring has been appointed a surgeon to St. Bartholomew's Hospital.

Mr. V. Warren Low, M.D., B.S., F.R.C.S., has been appointed surgeon to St. Mary's Hospital.

Mr. R. Miller, M.D., B.S., M.R.C.P., has been appointed assistant-physician to out-patients at St. Mary's.

Dr. John Howell Griffiths, M.D., B.S.(Lond.), has been appointed on probation for three months by the Metropolitan Asylums District as medical superintendent in the hospitals service, consequent on the death of Dr. Hume.

### Appointments Vacant.

Leeds General Infirmary.—House Physician.

Royal Albert Hospital, Devonport.—Resident Medical Officer.

Seamen's Hospital, Greenwich.—Senior House Surgeon and House Surgeon.

West Suffolk Education Committee.—Medical Inspector of School-children.

### The Question Box.

**SPECIAL NOTICE.**—Questions of interest affecting the honorary medical staff and hospital administration in all departments are invited. When any point arises we hope our readers will formulate it in a question and so co-operate to make this column of real value and interest. In this way the experience of the best-managed hospitals should be made helpful to the whole hospital world. We shall welcome the contribution of both questions and answers.

**N.B.**—The publication of an answer in this column does not necessarily preclude the publication of subsequent answers to the same question, for a question may involve points which require to be threshed out and fully discussed. Answers, if published, will be paid for at scale rates.

### THE HONORARY MEDICAL STAFF AND THE GENERAL COMMITTEE.

Question: Is it desirable that the honorary medical staff should have seats on the General Committee, or that they should meet as a Medical Board and recommend to the General Committee any changes they may consider necessary?

ANSWER: Have a Medical Board.

By Mr. F. Clare Melhado, Secretary Superintendent of the Middlesex Hospital.

I can only say that at this hospital the honorary medical staff are not represented upon the weekly Board of Governors, but meet as a Medical Committee and recommend to the Board any changes they may consider advisable. We find that this system works admirably.

### HOSPITAL ATTENDANCES OF THE HONORARY MEDICAL STAFF.

Question 1. Is it practicable to keep a book at each hospital in which the attending physicians and surgeons enter their name on arrival and departure?

ANSWER: Yes.

A London View.

By Dr. Habershon, M.D., F.R.C.P., Physician to the Brompton Hospital for Consumption.

It is the custom at Brompton Hospital to keep a book in the physicians' room in which the hour of attendance and departure is recorded. In the case of the assistant physician a book is brought to each assistant physician by the sister-in-charge of the out-patients' department, and he signs it in his room before leaving.

A Provincial View.

By Professor Saundby, M.D., C.M., F.R.C.P., Physician to the Birmingham General Hospital.

It is quite practicable.

A Scotch View.

ANSWER: Not the best way.

The practice of keeping a book registering the attendances of the honorary physicians and surgeons on their arrival and departure from a hospital would not generally find favour with the honorary staff, and where there is more than one entrance to the hospital buildings such a practice would be quite impracticable. The mere fact that a member of the staff visits the hospital at a certain hour, and remains a certain length of time, may convey quite a wrong impression of the amount of attention the patients under his care are receiving. If the board of managers, on the other hand, receive a weekly report of the number of operations performed by each individual surgeon, the length of time the patients were in hospital before the operation was performed, and the results of the operations, they would be in a better position to appreciate the relative importance of the work done by the various members of their staff. In the case of the physicians a weekly record of the number of admissions and the length of residence of each patient, with a note of the diseases of those who have been in residence for the longest period, might also be helpful; but the mere keeping of a register either by the porter at the gate, or by a clerk, or even by the signatures of the members of the staff individually seems to serve no particular purpose. The rules and regulations of some hospitals contain a clause to the effect that if a member of the staff finds it impossible to attend to his duties at the hospital he must notify the Secretary or responsible official to that effect, and a suitable deputy is arranged for. If such intimation is not given the matter should be immediately reported and dealt with.

Question 2. Is it allowable that the attendance book of the honorary medical staff shall be kept by the porter at the gate, who makes the entries and so saves the trouble to the visiting staff?

ANSWER: No.

By Professor Saundby, M.D., C.M., F.R.C.P., Physician to the Birmingham General Hospital.

It would be objectionable for the book to be kept by the porter. I am not expressing any opinion as to the desirability or need for such arrangements.

By Dr. Habershon, M.D., F.R.C.P., Physician to the Brompton Hospital for Consumption.

It would not be feasible, and would be undignified, to keep such a book in the porter's lodge. Besides, at most hospitals there are at least two if not three entrances to the hospital, and therefore the room assigned to the medical staff is the most convenient place.

### THE GRADUATES' MEDICAL SCHOOLS.

DIARY FOR THE WEEK, NOV. 29 to DEC. 4.

### ST. JOHN'S HOSPITAL FOR DISEASES OF THE SKIN, Leicester Square, W.C.

Chesterfield Lectures.—At the Hospital on Thursday evenings at 6 p.m., each lecture followed by clinical demonstrations:—

**Dec. 2, Baldness:** its Causes and Treatment.

### CENTRAL LONDON THROAT AND EAR HOSPITAL, Gray's Inn Road, W.C.

**At 4.30 p.m.—Dec. 2,** Mr. Stuart Low, **Cancer of the Throat: Its Prevention and Treatment.**

### THE HOSPITAL FOR SICK CHILDREN, Great Ormond Street, W.C.

**At 4 p.m.—Dec. 2,** Dr. Poynton, **Recurrent Family Periodic Jaundice.**

### NORTH-EAST LONDON POST-GRADUATE COLLEGE, Prince of Wales's General Hospital, Tottenham, N.

**At 4.30 p.m.—Nov. 30,** Dr. G. G. Macdonald, **New Methods for the Estimation of Sugar.**

### LONDON SCHOOL OF CLINICAL MEDICINE, Seamen's Hospital, Greenwich, S.E.

**At 3.15 p.m.—Nov. 29,** Mr. Wm. Turner, **Some Late Complications Following Operation.**

**At 2.15 p.m.—Nov. 30,** Professor Hewlett, **Some Practical Applications of Pathology to Clinical Medicine.**

**At 3.15 p.m.—Dec. 3,** Mr. McGavin, **Prostatic Enlargement and its Treatment.**

### THE POST-GRADUATE COLLEGE, West London Hospital, Hammersmith, W.

**At 10 a.m.—Nov. 29** and **Dec. 2,** Surgical Registrar, **Demonstration.**

**At 10 a.m.—Dec. 3,** Medical Registrar, **Demonstration.**

**At 12 noon.—Dec. 2,** Dr. Bernstein, **Pathological Demonstration.**

**At 12.15 p.m.—Nov. 30,** Dr. Pritchard, **Practical Medicine.**

**At 12.15 p.m.—Dec. 1** and **4,** Dr. Grainger Stewart, ditto.

**At 5 p.m.—Nov. 29,** Mr. Baldwin, **Practical Surgery.**

**At 5 p.m.—Nov. 30,** Dr. Pritchard, **Serum Diagnosis of Syphilis.**

**At 5 p.m.—Dec. 1,** Dr. Beddard, **Medicine—V.**

**At 5 p.m.—Dec. 2,** Mr. Edwards, **Clinical Lecture.**

**At 5 p.m.—Dec. 3,** Mr. Armour, **Head Injuries—II.**

### MEDICAL GRADUATES' COLLEGE AND POLYCLINIC, 22 Chenies Street, W.C.

**At 4 p.m.—Nov. 29,** Dr. J. M. H. MacLeod, **Skin.**

**At 5.15 p.m.—Nov. 29,** Mr. H. A. Ballance (Norwich), **Fractures of the Lower End of the Humerus in Children** (Illustrated).

**At 4 p.m.—Nov. 30,** Dr. J. W. Carr, **Medical.**

**At 5.15 p.m.—Nov. 30,** Mr. Harold J. Stiles (Edinburgh), **Treatment of Hernia in Infants and Young Children.**

**At 4 p.m.—Dec. 1,** Mr. Lenthal Cheatle, **Surgical.**

**At 5.15 p.m.—Dec. 1,** Mr. Charles Ryall, **Appendicitis in the Female.**

**At 4 p.m.—Dec. 2,** Sir Jonathan Hutchinson, **Surgical.**

**At 5.15 p.m.—Dec. 2,** Dr. David Drummond (Newcastle), **The Causes and Treatment of Indigestion.**

**At 4 p.m.—Dec. 3,** Mr. R. E. Bickerton, **Eye.**

### NATIONAL HOSPITAL FOR THE PARALYSED AND EPILEPTIC, Queen Sq., Bloomsbury, W.C.

**At 3.30 p.m.—Nov. 30,** Mr. Armour, **Osteoplastic Operations on the Skull.**

**At 3.30 p.m.—Dec. 3,** Dr. James Collier, **Diseases of the Brain Stem.**

### ROYAL SOCIETY OF MEDICINE, 20 Hanover Square, W.

**At 5 p.m.—Dec. 3, Laryngological Section:—**

Dr. Dundas Grant: (1) "Epithelioma of the palate and left pillar of the fauces." (2) "Secondary specific pharyngitis in young female."

Dr. W. H. Kelson: "Suppuration in frontal sinus and necrosis."

Mr. H. Betham Robinson: (1) "Left abductor paralysis in a young man—presumably toxic—from lead." (2) "Chronic laryngitis with curious appearance of right vocal cord."

Dr. R. H. Scanes Spicer: "Model to illustrate the variation in effect of costal and abdominal breathing on the throat."

And other cases.

**At 8.30 p.m.—Dec. 3, Section of Anæsthetics:—**

Mr. Bellamy Gardner and Dr. Salusbury Trevor: "Lymphatism with specimens and lantern illustrations."

**At 10 a.m.—Dec. 4, Otological Section:—**

Dr. Edward Law: "Deafness and discomfort in the right ear as early symptoms in a case of epithelioma originating near the right Eustachian tube."

Mr. Herbert Tilley: "A case of audible tinnitus."

Mr. W. Milligan: "Demonstration of Hayés pharyngoscope."

Mr. Richard Lake: "A short paper on the post-operative tests in eight cases of labyrinthine disease."

Dr. Albert Gray: "A contribution to the study of the pathology of deafmutism, with lantern-slide demonstration."

Mr. Sydney Scott: "Microscopical sections through the mastoid antrum in a fatal case of scarlet fever demonstrating *Streptococcus conglomeratus in sitû*. Brief notes of the case, with description of histological technique."

